# A Longitudinal Analysis of Naturalization and International Migration in Switzerland, 2011–2017

**DOI:** 10.1007/s12134-021-00857-7

**Published:** 2021-07-14

**Authors:** Juan Galeano, Aurélie Pont, Philippe Wanner

**Affiliations:** 1 nccr – on the move, Neuchâtel, Switzerland; 2 Geneva School of Social Sciences, Institute for Demography and Socioeconomics, University of Geneva, Geneva, Switzerland

**Keywords:** International migration, Naturalization, Dual citizenship, Subsequent migration, Switzerland

## Abstract

The notion of residential settlement associated with the acquisition of new citizenship has been recently challenged by a number of studies highlighting its instrumentality as a subsequent mobility factor. The long and diverse history of Switzerland as a country of immigration and the availability of rich data on naturalization and international migration that allow individuals to be followed over time make this country a valuable case for investigating the impact of naturalization on international (return or onward) migration. Using longitudinal data, we follow 88,900 immigrants who entered the country between 1998 and 2000 over a period of 84 months between January 2011 and December 2017, documenting changes in naturalization status and in migratory movements and their direction. Using different implementations of a Cox proportional hazards model, we examine whether and under what conditions the international migration behaviour of naturalized persons differs from that of non-naturalized persons. Our results show that the population accessing naturalization tends to be less mobile, but also that among third-country nationals, naturalization can trigger further international mobility, in particular among those with poor economic performance and with no family ties in Switzerland.

## Introduction

International migratory movement has been interpreted and analysed frequently and for many years as a unidirectional process. The classical conceptualization of migration as a binary link between the country of origin and the destination disregards the complexity of the phenomenon (Ahrens et al., 2016; de Hoon et al., 2019) by not considering, among others, key aspects such as return and/or onward migration (Borjas and Bratsberg, 1996; Cassarino, 2004). Within this simplistic framework, studies on naturalization have focused primarily on its consequences in terms of access to the labour market (DeVoretz and Pivnenko, 2005; for Switzerland, Fibbi et al., 2007; Bevelander and Pendakur, 2012), political participation (Just and Anderson, 2012), health (Minsart et al., 2013), social integration (Bloemraad et al., 2008; Hainmueller et al., 2017), and discrimination, with relatively few and only recent studies examining the relation between naturalization and subsequent international migration (Ahrens et al., 2016; Ramos et al., 2018; de Hoon et al., 2019). In the European case, this relationship is particularly relevant both in demographic and political terms given the asymmetry of intra-European mobility rights between citizens of UE/EFTA^1^ countries and third-country nationals (non-EU/EFTA).

Access to citizenship of the host country by the immigrant population is a recurring issue at the centre of political and public debates in Switzerland and many other Western countries. The majority of these debates focus on its scope and requirements, but they also reflect, more or less explicitly, underlying concerns about migrants’ motivation to undergo a naturalization process. However, empirical research evaluating the implications of naturalization for international migration remains scarce. In the case of naturalization and subsequent international migration, most of the recent research has approached this phenomenon from a qualitative perspective, with very few exceptions (Dronkers and Vink, 2012; de Hoon et al., 2019). This approach has decisively contributed to shed some light on the motivations, strategies, and experiences of various groups of non-EU migrants in their (re)emigration after accessing citizenship of a European country. However, the lack of reliable data on this subject in the vast majority of countries implies that neither the scope of the phenomenon has been yet approximated nor the socio-demographic factors involved explored from a quantitative perspective. The case of Switzerland, a country with a long diverse history of immigration and comprehensive data on emigration linked to the extinction of administrative (insurances and/or taxes) obligations, allows us to contribute to this body of research by providing an overview of different factors involved in the potential (re)emigration of naturalized migrants and to empirically inform debates on naturalization.

This paper interrogates the emigration behaviours of those individuals who become naturalized as Swiss citizens: Who access Swiss citizenship? Are there differences between migrants coming from EU/EFTA and non-EUEFTA countries? After accessing citizenship, do they remain in the country or move abroad? In other words, is the Swiss passport seen as a tool to increase mobility across borders? Are there differences in the re(emigration) behaviour of naturalized migrants related to the mobility opportunities attached to the passport of their prior nationality? What individual and/or household characteristics are associated with a greater propensity to (re)emigrate after accessing Swiss citizenship? Do household composition and economic performance influence (re)emigration behaviour? The paper’s main aim is to evaluate, from a quantitative perspective and within a longitudinal framework, whether and under what conditions the subsequent migration of naturalized persons living in Switzerland differs from that of non-naturalized persons, as well as between naturalized migrants from EU/EFTA and non-EU/EFTA countries.

## Literature Review and Research Hypothesis: Beyond the Settlement Assumption

Citizenship is usually conceived of as “a form of membership in a political and geographical community” (Bloemraad et al., 2008, p. 154), a unique and mutual relationship between an individual and the political community and within its borders. From a legal perspective, the legal status of citizens is granted by the state according to one or both parents’ nationality (*jus sanguinis*), the place of birth (*jus soli*), or a combination of both. Citizenship can also be requested by undertaking a naturalization procedure—in these cases, it is obtained via legal length of residence, marriage, historical ties, or a large investment in a country (Global Citizenship Observatory, 2019). When an application for nationality is based on the legal time of residence, such a request is traditionally interpreted as the expression of a strong willingness to settle and to integrate into the host country. In other words, it represents the *summum momentum* of the integration process, implicitly indicating sedentarism in the host society. However, in a world characterized by global and transnational inequalities (Shamir, 2005; Beck, 2007) where international mobility continues to be a “scarce” resource for the vast majority of the population (Bauman, 2002), passports and feelings of attachment related to citizenship have proven to not necessarily go together (Coutin, 2003; Harpaz, 2013; Szewczyk, 2016; Ramos et al., 2018). Furthermore, considering that passports currently have uneven value (Castles, 2005; Harpaz, 2019b), they do not allow access to the same rights and opportunities, whether inside or outside the country of issuance (Kochenov and Lindeboom, 2017). Hence, hierarchization of the right to migrate, as outlined by Castles (2005), implies that mobility opportunities are distributed unequally, depending on the nationality held (Shachar, 2009; Mau, 2010).

The propensity to naturalize is affected by both migrants’ individual characteristics and the social context in the destination country. In the case of social environment, the positive correlation between the ethnic community size and naturalization has been documented (Yang, 1994; Logan et al., 2012). In this sense, larger co-ethnic communities facilitate naturalization as they provide information related to the procedures, benefits, and experiences to its members. No less important than individual or contextual characteristics are the reasons of migrants to engage in a naturalization process. As highlighted by recent and primarily qualitative research on this subject, the motivations and strategies behind naturalization vary. The acquisition of citizenship could be motivated by the desire to stabilize the place of residence (Finotelli et al., 2018), and/or it can be pursued as a protection mechanism against the bureaucratic apparatus of the state (Graeber, 2016; Aptekar, 2016; Della Puppa and Sredanovic, 2017; Ramos et al., 2018). Moreover, naturalization in any member of the European Union (EU) or the European Free Trade Association (EFTA) makes accessible a number of additional rights, including the free movement of persons in the EU context^2^, thus promoting opportunities for transnational and international mobility (Ahrens et al., 2016; Ramos et al., 2018; Ortensi and di Belgiojoso, 2018; de Hoon et al., 2019). Hence, due to the additional benefits attached to the acquisition of Swiss citizenship for a non-EU/EFTA person, *naturalization rates are expected to be higher for non-EU/EFTA individuals than they are for the EU/EFTA population (H1).*

During the last decades of the twentieth century, many European countries changed their laws to allow, recognize, or at least tolerate the potential pluri-nationality of their citizens (Blatter, 2011; Harpaz, 2019a). The aforementioned unequal distribution of rights associated with a nationality, in a context of growing international mobility (and inequality), has led some scholars to speak of “instrumental” (Ip et al., 1997; Aguilar Jr., 1999), “compensatory” (Harpaz, 2019b), or “strategic” citizenship (Finotelli et al., 2018; Harpaz and Mateos, 2019). These conceptual frameworks aim to challenge the settlement or “sedentarist” assumption (Sheller and Urry, 2006; Halfacree, 2012) underlying the vast majority of studies, policies, and even the popular imaginary about naturalization, mainly associated with permanent or long-term residential settlement in the host country. They also recognize that accessing the citizenship of the host country does not necessarily imply an increased feeling of belonging. In doing so, this body of research focuses on the rights and opportunities involved in obtaining a more valuable nationality than the original. Research on this topic also highlights how, for third-country nationals, obtaining the legal status of an EU citizen is seen as a safeguard for intra-European mobility in case of economic crisis (Della Puppa and Sredanovic, 2017; Mas Giralt, 2017; Ramos et al., 2018). Thus, naturalization increases “mobility capital” by constituting a step forward in the migration trajectory through the creation of mobility opportunities transformable into personal or family advantage and by permitting one to choose to remain immobile (Moret, 2017, p. 5). Both onward and return migration can result from naturalization, as well as other varied “mobility outcomes”, such as circular migration, holidays, or family visits (de Hoon et al., 2019). A country such as Switzerland, with a high proportion of foreign nationals from both EU/EFTA and non-EU/EFTA countries, constitutes an ideal case to investigate the strategic dimension of access to citizenship. In this sense, *we expect the risk of (re)emigration among the naturalized population to be higher for the non-EU/EFTA group than for those from EU/EFTA countries (H2).*

Within migration studies, the (re)emigration of migrants is a topic that has attracted considerable attention from scholars over the past decades (for a comprehensive review see, Nebky, 2006). While much of that work has focused on return migration, onward migration, particularly within the EU27, has also gained prominence in recent years (Nebky, 2006; Ortensi and di Belgiojoso, 2018; Monti, 2020). Both types of (re)emigration have been largely explained following neoclassical economic or new economics of labour migration postulates. Among the many causes to which return migration has been attributed, in the short term, is the failure of the migration project (Constant and Massey, 2003). In the long term, it has been proposed that return migration may respond to an optimal residential location plan linked to the life cycle of migrants (Borjas and Bratsberg, 1996). In this direction, it has been documented that retirement increases the probability of return migration (Constant and Massey, 2003; Kuhlenkasper and Steinhardt, 2017). Onward migration, instead, would be most commonly linked to labour market opportunities and the intention to better match skills and employment in the new destination country (Nebky, 2006; Kuhlenkasper and Steinhardt, 2017; Ramos et al., 2018). Among the different factors identified as responsible for the mismatch between skills and employment, discrimination is particularly relevant since it does not distinguish between naturalized and non-naturalized migrants. In the Swiss case, there is recent evidence of discrimination on the basis of ethnic origin in hiring decisions (Zschirnt, 2020; see also Fibbi et al., 2003). Therefore, besides personal aspirations, there is also a nexus between experiences in the host country and especially feeling discriminated and emigration (Kunuroglu et al., 2018; Sener 2019). In summary, if aspirations behind migration are not met or experiences are not satisfactory enough in the first country of destination, then re-emigration will be considered, reinforcing self-selectivity of migration (Nebky, 2006; Kuhlenkasper and Steinhardt, 2017; Monti, 2020). Considering the different set of motivations behind each type of migration and the differences in the socio-demographic profiles between the two groups, *we expect that, after naturalization, onward migration will be more common among the non-EU/ EFTA population (H3).*

In both cases, it has also been acknowledged that family ties (spouse or children) in the host country discourage (re)emigration. In Switzerland, Steiner (2019) observes that few immigrants who arrived because of family formation declare to have intentions of (re)migrating in the future, while many of them intend to naturalize. In her study about (re)emigration in Sweden, Monti (2020) shows that married individuals tend to re-emigrate less than singles and that the likelihood of (re)emigration increases with the number of children, except if at least one child is born in Sweden. In the case of Denmark, Jensen and Pedersen (2008) also highlight the diminishing effect of the number of children over (re)emigration and report that an immigrant married to a Danish citizen is less likely to leave. Although the literature on the (re)emigration of migrants is abundant, works devoted to the (re)emigration of naturalized migrants are scarce. Reasons for the international relocation of recently naturalized persons were investigated by Ahrens et al. (2016), who report different motivations related to the labour market, education opportunities, discrimination, and/or racism, among other causes, as the explanatory mechanisms underlying second migration. In the case of the Netherlands and based on longitudinal data, de Hoon et al. (2019) show that accessing Dutch citizenship increases the likelihood of subsequent migration for some groups of refugees, particularly those with poor integration into the labour market. Thus, the last hypothesis we want to address is that *household size and economic position are negatively associated with the propensity to engage in subsequent international migration after naturalization (H4).*

## A Brief Overview of Citizenship Acquisition in Switzerland

Passports simultaneously reflect the opportunities available inside and outside the country of issuance. From this perspective, the Swiss passport is relatively strong (Kochenov and Lindeboom, 2017). However, in this *jus sanguinis* country, acquiring it other than through blood ties is challenging; its policy regarding access to nationality ranks in the Migrant Integration Policy Index (MIPEX) as one of the most restrictive in Western Europe (MIPEX, 2019). Citizenship acquisition is also regularly at the centre of heated mass media and political debates in this country. Between 1922 and 2017, Swiss citizens were asked to vote eight times on issues related to citizenship and nationality, including three popular initiatives with a restrictive vocation (Arrighi, 2017). For instance, in 2008, the Swiss citizens had to vote on a popular initiative launched by a right-wing party that calls “to end mass naturalizations”.

Citizenship acquisition in Switzerland is not granted automatically depending on length of residence but is instead based on individual decision-making driven by the recommendations of members of the administration in the form of a process of naturalization. Two acquisition modes are made available by the law, depending on the personal situation of each candidate. The main one, ordinary naturalization, potentially applies to all individuals with foreign nationality (or stateless persons) living in Switzerland and meeting certain requirements^3^ related mainly to their length of stay (12 years at the federal level), social integration^4^, lack of a criminal record, and financial independence (Gutzwiller, 2008). For foreigners who were born in Switzerland or arrived there as children, the years between the ages of ten and twenty count double, which can contribute to reducing the waiting period for eligibility to apply for Swiss citizenship. Once these criteria are met, the acceptance rate is generally very high. A recent research based on 20 cantons reports around 90% of positive decisions (Probst et al., 2019). To the authors’ knowledge, there are no recent studies on rejection reasons. However, certain forms of decision-making on naturalization applications, in particular closed ballot voting (which are not allowed since 2003), have resulted in discriminatory practices against candidates from the former Yugoslavia and Turkey (see Helbling, 2008; Hainmueller and Hangartner, 2013). The other mode, facilitated naturalization, concerns the legal partner of a Swiss citizen (after three years of conjugal community) who has lived in Switzerland for 5 years. Children of naturalized persons (not included in the application when they were minors) are also subject to this acquisition mode. Thus, the criteria related to the length of stay differ between these two regimes.

The procedures and decision-making levels also vary; responsibilities are divided among the three administrative levels (municipalities, cantons, and the Confederation) for ordinary naturalization, while the federal administration handles the facilitated procedure. Therefore, the acquisition of citizenship is highly decentralized, which is emblematic of Swiss federalism. This grants a relatively wide margin of discretion and freedom of action to lower administrative levels, who are in charge of evaluating naturalization applications (including the level of integration). For instance, the length of residence under any types of residence permit should be counted. However, it has been shown (Probst et al., 2019) that in the past, several cantons set higher requirements and did not consider the length of stay under certain types of permits (particularly for people arriving through asylum). For clarification and harmonization purposes, the law on naturalization changed at the federal level in 2018. The main modifications involve, among others, the reduction of the period of residence from 12 to 10 years and the requirement to possess an establishment permit (C permit) to apply for naturalization. It also defines more precisely the integration criteria necessary for granting naturalization; for instance, a level of oral and written language skills certified and no social assistance must have been received during the 3 years preceding the application. This law is supposed to lead to a tightening of the conditions for naturalization because the cantons whose practices were more flexible are henceforth obliged to adapt their legislation to the new federal law (Probst et al., 2019, p.133).

Between 2011 and 2017, more than 200,000 naturalizations were registered in Switzerland (Table 5, Appendix). Descriptive results show that among those who gained access to Swiss citizenship in 2011, more than 2000 emigrated (at least one time, but sometimes more than one time), yielding a 6.2% emigration rate for an almost 7-year period.

## Data and methodology

Data on naturalization and international migration from 2011 to 2017 are available through the Swiss population register. Based on the available data, we established a longitudinal dataset to test whether the international migration of naturalized migrants differs from that of non-naturalized migrants and to describe the differences in emigration behaviours according to the citizenship of the country of origin. For this paper, the population register was also linked to another administrative register, the register of the first pillar, which provides the yearly income of every worker in Switzerland. The process of creating the longitudinal dataset can be summarized as follows:

Considering that prior to 2017, ordinary naturalization in Switzerland implied permanent residence for 12 years, and considering that data on emigration among the Swiss population (naturalized citizens) is available from 2011 onwards, we define the population under analysis as those entering the country between 1998 and 2000 with foreign citizenship, who were still in Switzerland at the end of 2010 and who were aged 18 or more years by that time. The latter criterion ensures that the process of naturalization acquisition was initiated by each individual and not by the person’s parents. This procedure yields a total of 88,900 people. A crucial feature of our dataset is the availability of the naturalization and/or the international migration date (if these events occur). Thus, we track monthly changes in both events between January 2011 and December 2017 (84 months) for each individual. In those very few cases in our dataset in which naturalization and emigration took place in the same month, we lag the latter by 1 month. When emigration occurs, the country of destination is also recorded if it is declared to the authorities when leaving Switzerland, which is not always the case. In total, 15% of emigrants did not give information about the destination country; 62% returned to their country of first nationality (return migration), and 23% emigrated to a country other than their country of origin (onward migration).

We fit Cox proportional hazards models to examine the occurrence of international migration over a period of 84 months. Event history models of this type allow us to examine both the timing and occurrence of an event, permitting the inclusion of both time-dependent and time-invariant covariates (Cox, 1972; Fox and Weisberg, 2011; Therneau et al., 2019). The measure of interest obtained from a Cox regression model is the hazard ratio (HR). The HR represents the ratio of hazards between two groups at any particular point in time. In our study, a HR < 1 indicates a reduced hazard of emigration compared with the reference category, whereas a HR > 1 indicates an increased hazard of (e)migration.

To test the conditions under which naturalization is likely to be followed by international (e)migration, we added a set of covariates described in the next section. In addition to the general models measuring emigration as a single event, we fit three competing outcome models to evaluate possible variations depending on the direction of the movement (onward migration, return migration, and undeclared destination). Due to the abovementioned differences in freedom of movement within the EU between holders of different passports, we run, in all cases, separate models for two different population groups: EU/EFTA and non-EU/EFTA.

### Covariates Included in the Model

We measure emigration and naturalization as time-dependent variables, and we track monthly changes between January 2011 and December 2017 (84 months). Naturalization is measured as a dichotomous variable (non-naturalized vs naturalized). Within the set of socio-demographic characteristics, we include sex, age (as registered in 2011), and the country of the previous nationality grouped as either EU/EFTA or non-EU/EFTA. We include information on household size as recorded in 2011 in four categories (1 person, 2 persons, 3 persons or 4 or more persons) as a way to proxy family composition. The socio-economic position of migrants is introduced as a categorical variable using the quartile distribution of perceived total income between 2005 and 2010. To assess the influence of the previous international migration or internal mobility trajectory(ies), we include two different dichotomous variables: one classifying the population as those who performed an international emigration between 2005 and 2010 and those who did not and the other classifying the population as those who performed 2 or fewer inter-cantonal movements between 2005 and 2010 and those who moved 3 or more times. In addition to these individual characteristics, two other covariates concerning the origin group are included and controlled for in the model: the number of co-nationals living in Switzerland in 2014 and the Nationality Quality Index (NQI; Kochenov and Lindeboom, 2017) as recorded in 2012. The NQI measures the freedom of movement and settlement attached to a certain passport, and it is included in the models as a categorical variable defined by the quartiles of its distribution.

## Descriptive Analysis of Naturalization and Emigration

**Fig. 1:**
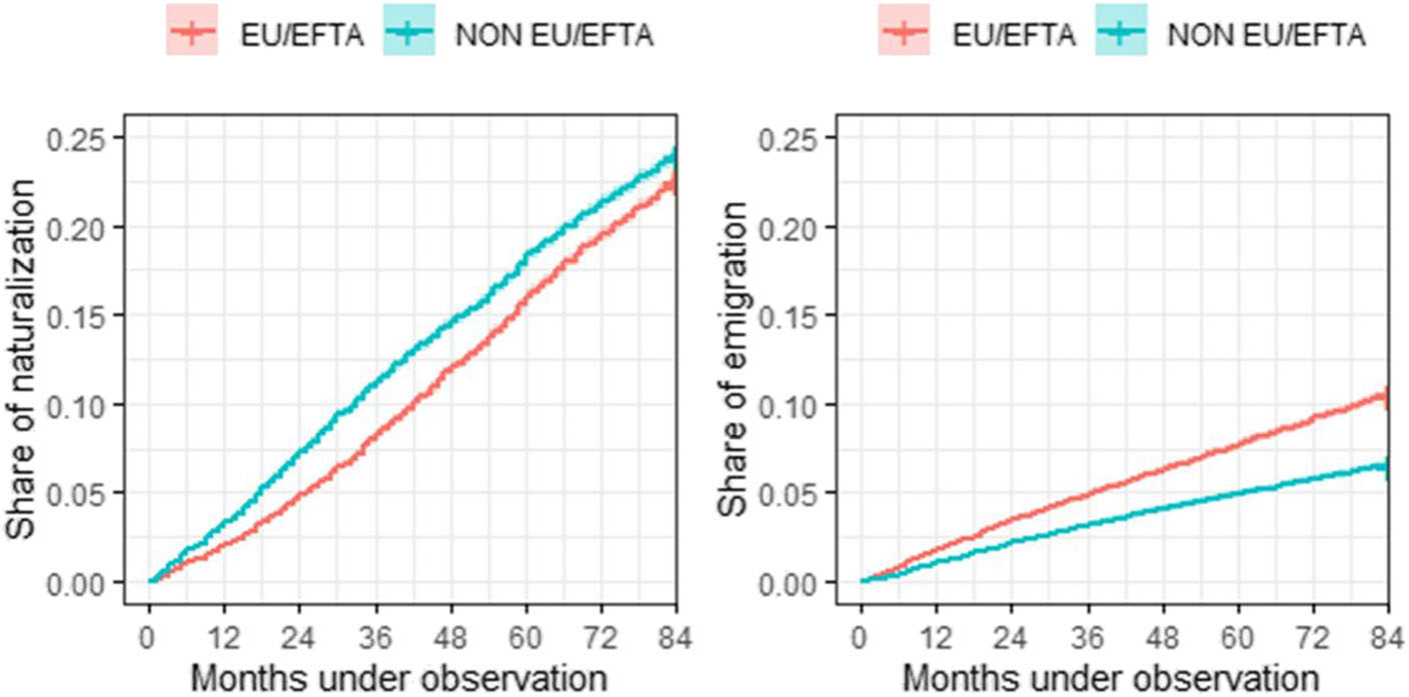
Kaplan-Meier cumulative incidence of naturalization and emigration by groups, January 2011–December 2017. Source: Own elaboration based on data from the Swiss Statistical Office

As stated above, our dataset contains information for 88,900 individuals. Almost half of them (49%) are EU/EFTA citizens (75% have German, Portuguese, French, or Italian citizenship), and the other half (51%) are non-EU/EFTA citizens. As a consequence of the high share of arrivals of people from the Balkan countries between 1998 and 2000 (during the final years of the Yugoslav Wars), the non-EU/EFTA group is composed mostly of Serbians (21%), Macedonians, Kosovars, and Turks (27%). Between 2011 and 2017, almost one in four (23.3%, 20,689 people) individuals in our dataset received Swiss nationality (Table 6, Appendix), with a similar naturalization rate between groups: 22.5% for EU/EFTA migrants and 24% for non-EU/EFTA migrants (Fig. 1). However, these aggregated results hide important differences between nationals of different countries in their access to Swiss citizenship. Fewer than one in 10 individuals with Portuguese or Austrian citizenship and between two and three in 10 individuals with French or German citizenship were naturalized between 2011 and 2017, but more than four in ten Romanian, Bulgarian, Iranian and Russian citizens were naturalized in this period.

Regarding international migration, our dataset records 7547 emigrations between 2011 and 2017 (an 8.5% cumulative emigration rate). In this case, differences between the two population groups are more pronounced. The cumulative emigration rate of 10.5% in the EU/EFTA population is almost double that in the non-EU/EFTA population (6.5%). The data also suggest that the gap between the two groups widens over time. The differences in the emigration rates between the two groups under analysis are probably rooted in the more advantageous conditions for intra-European mobility enjoyed by the former group than the latter.

### Naturalization as a Latent State: a Univariate Cox Model

Access to the nationality of the host country is by no means the result of a 1-day action or decision. In contrast, it is a process that, as we have seen in the Swiss case, requires the proactive attitude of the applicant and the fulfilment of numerous requirements. Naturalization, then, may be considered the end point of a decision that, more or less consciously, has been made several years before: a latent state. To test this hypothesis in the Swiss case, we compute two separate univariate Cox regression models (one for each population group) considering naturalization as a time-invariant covariate.

**Fig. 2:**
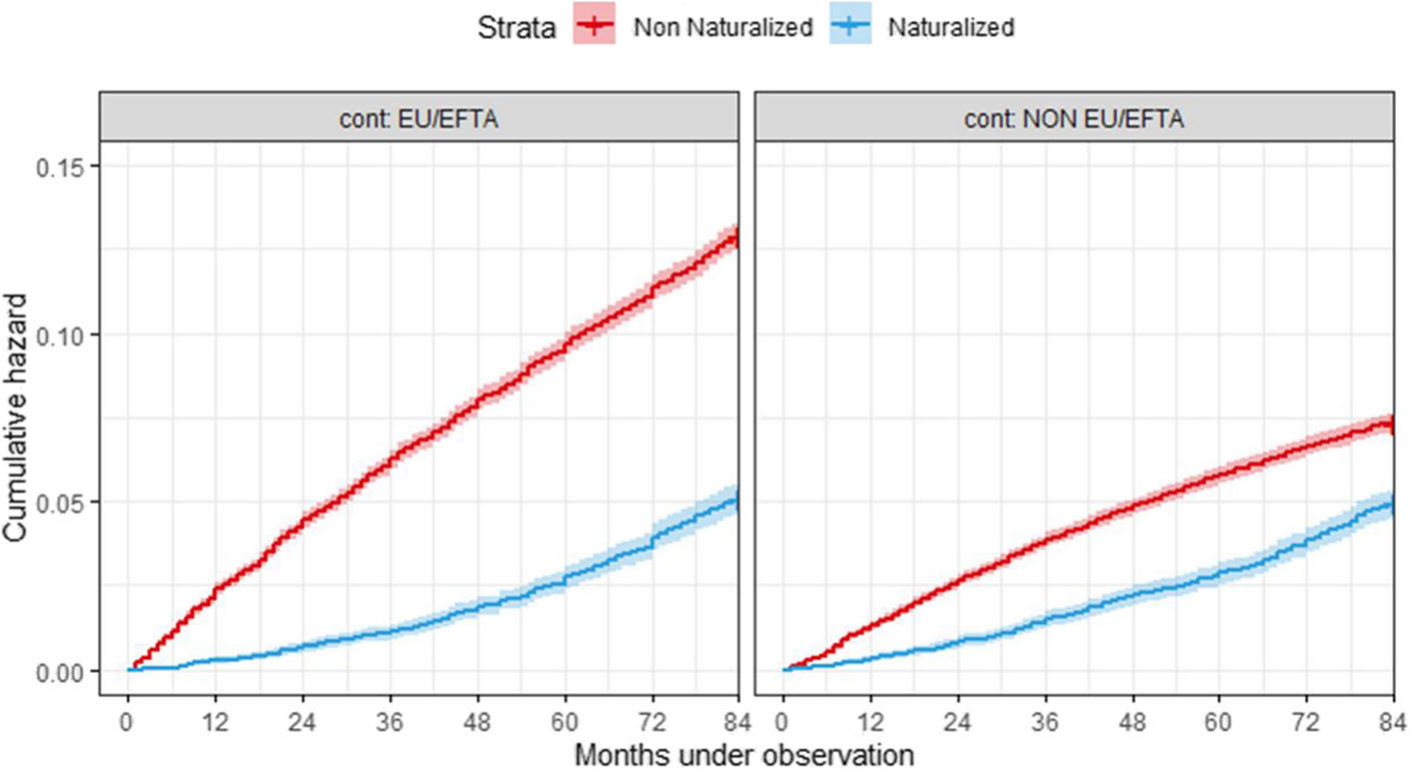
Cumulative hazard of emigration by month and population group. Source: Own elaboration based on data from the Swiss Statistical Office

As reflected in Table 1, the HR of emigration for the population acquiring Swiss citizenship between 2011 and 2017 for either of the two population groups under analysis (EU/EFTA or non-EU/EFTA) is lower than that of the non-naturalized population. In addition to the abovementioned differences in the occurrence of emigration between groups (also reflected in Fig. 2), this first analysis reveals that individuals who will become naturalized tend to be less internationally mobile than their non-naturalized counterparts and that differences in the hazard of emigration are greater for the EU/EFTA population: 61% lower versus 33% lower for the non-EU/EFTA group. However, this first overview also suggests that among migrants from non-EU/EFTA countries, the acquisition of Swiss nationality concerns individuals who, in addition to other benefits attached to citizenship, are also looking to find a way to facilitate their international mobility (H2). In both cases, the emigration HRs in this first univariate analysis reflect the lower propensity to move of the population accessing or willing to access Swiss citizenship, which is somewhat expected considering the highly restrictive access to nationality imposed by the different administrative levels of the Swiss government compared to the access policies in neighbouring EU countries.

**Table 1:** Cox model considering naturalization as a fixed covariate

	EU/EFTA			NON-EU/EFTA		
Characteristic	Hazard ratio	95% CI	p-value	Hazard ratio	95% CI	p-value
Naturalization						
No (Ref.)	--	--		--	--	
Yes	0.39	0.36–0.43	<0.001	1.46	0.61–0.74	<0.001
	N = 43,340 Events = 4562			N = 45,560 Events = 2985		

Source: Own elaboration based on data from the Swiss Statistical Office

However, one of the main questions we want to address in this paper is related to the potential of naturalization as a mobility trigger. To do so, it is necessary to fit the Cox model while considering naturalization as a time-dependent variable. The “citizenship to go” or “citizenship as a ticket to mobility” hypothesis has already been explored from both a qualitative (Della Puppa and Sredanovic, 2017) and a quantitative perspective (de Hoon et al., 2019), respectively, for Italy and the Netherlands. Differences in the analytical strategy significantly affect the results obtained in terms of elucidating to what extent and under what circumstances the acquisition of Swiss nationality functions as a factor that impacts the potential subsequent international migration of those who receive it. Table 2 shows the results of a univariate model including naturalization only. Once naturalization is added to the model as a time-dependent variable, two major changes occur. First, the difference in the hazard of emigration between those who naturalize and those who do not (H2) for the EU/EFTA population almost vanishes (HR: 0.91) and loses statistical significance (p-value: 0.074). Second, for the non-EU/EFTA population, the hazard of emigration changes signs and becomes positive. For this population group, accessing Swiss citizenship increases the HR of international emigration by 46%. The next section addresses the potential triggers of emigration after naturalization.

**Table 2:** Cox model considering naturalization as a time-dependent covariate

Model return	EU/EFTA			NON-EU/EFTA		
Characteristic	Hazard ratio	95% CI	p-value	Hazard ratio	95% CI	p-value
Naturalization						
No (Ref.)	1	--		1	--	
Yes	0.91	0.82–1.01	0.074	1.46	1.32–1.62	<0.001
	N = 53,012 Events = 4562			N = 56,339 Events = 2985		

Source: Own elaboration based on data from the Swiss Statistical Office

### Triggers of Post-naturalization International Emigration

After controlling for a set of socio-demographic characteristics, we find a change in sign for the emigration HR of the naturalized EU/EFTA population, which continues to be close to zero but without statistical significance (p-value=0.3). However, the results for the non-EU/EFTA population provide interesting insights into the triggers of post-naturalization international emigration and accentuate the previously described positive association (Table 3).

**Table 3:** Cox proportional hazards model for the risk of emigration with covariates

Model 2	EU/EFTA			NON-EU/EFTA		
Characteristic	Hazard ratio	95% CI	p-value	Hazard ratio	95% CI	p-value
Naturalization						
No (Ref.)	1	--		1	--	
Yes	1.05	0.95–1.17	0.3	1.64	1.47–1.82	<0.001
Sex						
Male (Ref.)	1	--		1	--	
Female	0.78	0.73–0.83	<0.001	0.58	0.54–0.63	<0.001
Age						
Age in 2011 squared	1	1–1	<0.001	1	1–1	<0.001
Household size	1	--		1	--	
1 person (Ref.)						
2 persons	0.87	0.80–0.94	<0.001	0.88	0.79–0.97	0.015
3 persons	0.66	0.60–0.72	<0.001	0.63	0.56–0.71	<0.001
4 or more	0.57	0.52–0.62	<0.001	0.44	0.40–0.49	<0.001
Income						
1st quantile (Ref.)	1	--		1	--	
2nd quantile	0.74	0.66–0.82	<0.001	0.67	0.61–0.74	<0.001
3rd quantile	0.60	0.54–0.67	<0.001	0.56	0.51–0.63	<0.001
4th quantile	0.57	0.52–0.62	<0.001	0.46	0.41–0.51	<0.001
Inter-cantonal m						
Fewer than 2 (Ref.)	1	--		1	--	
3 or more	1.60	1.07–2.39	0.043	2.69	1.48–4.86	0.001
International m						
No (Ref.)	1	--		1	--	
Yes	1.02	0.75–1.45	0.8	0.61	0.53–0.70	<0.001
Co-nationals (x10k)	0.98	0.98–0.98	<0.001	0.87	0.86–0.89	<0.001
NQI						
4th quantile (Ref.)	1	--		1	--	
3rd quantile	0.60	0.47–0.76	<0.001	0.58	0.52–0.66	<0.001
2nd quantile	--	--		0.59	0.53–0.65	<0.001
1st quantile	--	--		0.40	0.35–0.45	<0.001
	N = 52,312			N = 55,433		
	Events = 4,438			Events = 2,882		

Source: Own elaboration based on data from the Swiss Statistical Office

First, regarding sex, the second model (Table 3) shows that women have a lower hazard of emigration than men (41%, or 1.69 times lower, in the case of the non-EU/EFTA population). Although we do not have information on the composition of the families of the individuals in our dataset, we do have the number of people in each household, which serves as an approximation of the household type. Thus, household size has an inverse relation with the risk of leaving the country after obtaining a Swiss passport (H4). The larger the size of the household size, the lower the HR of subsequent emigration, illustrating the retaining effect of family. Income also has a negative relation with emigration and a powerful retaining effect; the higher the annual income is, the lower the hazard of leaving the country (H4). For instance, an individual in the 4th quantile of the income distribution has a HR of emigration 52% lower than someone in the first. Residential stability, approximated here as the number of inter-cantonal migrations performed between 2005 and 2010, has a major impact on the propensity to emigrate of the naturalized population (and of the non-naturalized population). Individuals who have experienced 3 or more inter-cantonal movements between 2005 and 2010 exhibit a HR of emigration 2.6 times higher than that of those who have experienced 2 or fewer (H3). In contrast, those who migrated internationally at least one time during the same period (2005–2010) had a 39% lower hazard than those who had not. To evaluate the impact of the network of the country of origin, we include a covariate in the model for the number of co-nationals (in tens of thousands) living in Switzerland in 2014. As in previous research (de Hoon et al., 2019), the result suggests that country-of-origin networks also have a retaining effect, as there is a 13% lower hazard of international emigration per 10 thousand co-nationals. Finally, the strength of the previous nationality in terms of how many countries an individual can access without a visa, measured by the value of the NQI, seems to be positively associated with emigration, and lower HRs of emigration are observed for those previously holding the weakest passports.

### Moving Forward or Coming Back Home?

As the destination country for those who leave Switzerland is also available in our dataset, we fit three competing outcome models to evaluate variations in the previous results depending on the direction of the movement (Table 4, return migration, onward migration, and undeclared). Although we present only the HRs for naturalization, in these models, we control for the same set of covariates as in Table 3. These three models are not directly comparable in terms of their size, as they have been computed separately, but the HRs can be evaluated according to their direction and significance.

**Table 4:** Competing destination models

Model return	EU/EFTA			NON-EU/EFTA		
Characteristic	Hazard ratio	95% CI	p-value	Hazard ratio	95% CI	p-value
Naturalization						
No (Ref.)	1	--		1	--	
Yes	0.8	0.70–0.93	0.003	1.21	1.04–1.40	0.012
Other controls	Yes			Yes		
	N = 52,312			N = 55,433		
	Events = 3101			Events = 1872		
Model onward	EU/EFTA			NON-EU/EFTA		
Characteristic	Hazard ratio	95% CI	p-value	Hazard ratio	95% CI	p-value
Naturalization						
No (Ref.)	1	--		1	--	
Yes	2.14	1.83–2.49	<0.001	3.58	3.03–4.22	<0.001
Other controls	Yes			Yes		
	N = 52,312			N = 55,433		
	Events = 1411			Events = 768		
Model unknown	EU/EFTA			NON-EU/EFTA		
Characteristic	Hazard ratio	95% CI	p-value	Hazard ratio	95% CI	p-value
Naturalization						
No (Ref.)	1	--		1	--	
Yes	0.37	0.18–0.76	0.007	0.58	0.41–0.83	0.003
Other controls	Yes			Yes		
	N = 52,312			N = 55,433		
	Events = 354			Events = 710		

Source: Own elaboration based on data from the Swiss Statistical Office

In general, the results presented above are confirmed for the non-EU/EFTA population when we consider only return migration (to the country of the previous nationality). In the case of the EU/EFTA population, modelling migration considering the destination results in a significantly lower hazard of return migration for the naturalized population (20% lower). The acquisition of Swiss citizenship seems to significantly enhance the hazard of migration for both population groups when naturalized persons move onward to another country different from the country of the previous nationality but with a higher intensity for the non-EU/EFTA group (H2). Almost half of the naturalized persons in both population groups who emigrated after obtaining Swiss nationality (476 individuals) moved onward. France is one of the most popular destinations for both population groups, as 23% of the non-EU/EFTA naturalized population and 15% of the naturalized EU/EFTA who left the country migrated to France. This is probably the result of the imbalance between the housing markets in Switzerland and France, with lower prices on the latter. Once naturalized, persons of a foreign origin can live in French regions and continue working in Switzerland. For instance, it is interesting to observe that among those naturalized between 2011 and 2016 who left Switzerland to go to France, 66% still received earnings from Switzerland in 2017 (the rate is 23% among those not naturalized) according to the income data provided by the National Statistical Office. After France, the UK and Germany rank as top destinations for non-EU/EFTA persons, and the USA, the UK, and Germany rank as top destinations for the EU/EFTA group (Table 7, Appendix). For the non-naturalized EU/EFTA migrants leaving Switzerland between 2011 and 2017, France, the USA, Germany, and the UK were also the favourite destinations. For the non-EU/EFTA group, Kosovo ranks first, followed by the USA and France. However, all members of the non-naturalized population emigrating from Switzerland to Kosovo entered the country with a Serbian passport at the end of the Balkan Wars. Thus, their movement may not be considered onward migration. Finally, the acquisition of Swiss citizenship reduces the hazard of leaving the country without formal notification, as illustrated by the HRs of emigration for the naturalized population when the destination is unknown. In this case, the intensity of the effect is higher for the EU/EFTA group.

## Concluding Remarks

A growing body of research has recently challenged the implicit “sedentary assumption” (Sheller and Urry, 2006; Halfacree, 2012) underlying the vast majority of studies on the multiple outcomes of naturalization, particularly those related to international migration. In doing so, one of the obstacles researchers face in quantitatively addressing the scope of the phenomenon and its determinants is the lack of reliable data on emigration. In this sense, Switzerland is one of the few European countries with a complete register of population, which records the emigration of legal immigrants and provides the necessary data infrastructure to overcome this issue.

The inequality of rights and opportunities associated with nationality has led a number of scholars to investigate the strategic dimension of accessing multiple citizenship (Finotelli et al., 2018; Harpaz and Mateos, 2019). Before analysing the impact of naturalization on international migration, the data allowed us to evaluate the compensatory dimension of citizenship proposed by Harpaz (2019b). We find some evidence, in the form of a slightly higher cumulative rate of naturalization, supporting the hypothesis that naturalization is more appealing for the non-EU/EFTA group, as also reported by Monti (2020) in the case of Sweden. For this population, accessing Swiss nationality compensates for some of the limitations of their original nationality, particularly those associated with intra-European mobility. Hence, in general terms, it is important to highlight that aside other positive outcomes, naturalization contributes to reduce the unequal distribution of “mobility capital” resulting from the “birthright lottery” (Shachar, 2009) among the population living in Switzerland. However, policy-wise, it is important to highlight that differences in naturalization rates between immigrant groups are much more pronounced when computed based on the country of first nationality, either within the EU/EFTA or within non-EU/EFTA groups. Thus, they must be discussed after considering all the symbolic and practical factors behind the decision to undergo a naturalization process (see, for instance, Loretan and Wanner, 2017).

The conceptualization of access to a new citizenship as instrumental (Ip et al., 1997; Aguilar Jr., 1999) can lead to a misinterpretation of the scope of the phenomenon in regard to subsequent migration. Citizenship acquisition, even conceived as a latent state, exerts a general retaining effect. During the period of analysis, the naturalized population (re)emigrated less than the non-naturalized population in both groups, although intra-group differences are smaller for the non-EU/EFTA population. This expected outcome may be reinforced in the Swiss case by the highly restrictive access granted by Swiss authorities to citizenship. However, the strategic dimension citizenship in relation to (re)emigration becomes apparent when naturalization and subsequent migration are modelled as time-dependent variables. After controlling for different confounding factors in the medium term, our analysis reveals an increased likelihood of (re)emigration after naturalization for the non-EU/EFTA population. This evidence suggests that events are deliberately ordered by a significant part of this population group; first they access to naturalization and then migrate.

When the direction of the movement is considered, we find that half of the naturalized population in our dataset who left Switzerland between 2011 and 2017 moved to a different country from the country of their previous nationality (onward migration). While the acquisition of Swiss nationality seems to heighten the hazard of onward migration, the intensity of the association is higher among the non-EU/EFTA population. However, the different meaning of obtaining Swiss citizenship between the population groups is clearly reflected in relation to return migration. For EU/EFTA migrants, Swiss citizenship is not as much a means of further mobility, leading to a lower return migration likelihood. The possible different set of motivations that drives the decision regarding onward or return migration for members of the two population groups needs further qualitative research to enrich policy design and debates on naturalization. Data also reveal that citizenship acquisition has a demonstrable administrative impact, decreasing the risk of leaving the country without a formal notification of those who become naturalized. This can be explained by the fact that naturalized citizens will probably and more frequently leave the door open for a return to Switzerland than non-naturalized foreigners. They therefore announce their departure to be in line with the formal requirements of Switzerland in terms of insurance or taxes.

Holding citizenship of the host country does neither guarantee the same rights and opportunities as native-born nor protect against discrimination (Zschirnt, 2020), which can motivate emigration (Kunuroglu et al., 2018; Sener 2019). Our results highlight relevant socio-demographic and contextual characteristics that are associated with a higher chance of subsequent migration after obtaining Swiss citizenship. Previous research (Nebky, 2006; Kuhlenkasper and Steinhardt, 2017; de Hoon et al., 2019; Monti, 2020) on (re)emigration has highlighted poor integration into the labour market as one of the key triggers of onward migration. Although we lack specific information on this topic in our dataset, we approximate labour market incorporation through annual income and find a negative relation between earnings and the risk of (re)emigration. Thus, the population with lower income is exposed to a higher risk of (re)emigration after naturalization. However, in terms of the policy implications of this finding, it would be necessary to explore the hypothesis that in some Swiss agglomerations, such as Geneva or Basel, part of the population with lower income who access citizenship may be moving to border areas of France as a coping strategy to deal with higher housing market prices in Switzerland while continuing to work in the country. Related to poor labour market incorporation, our results also highlight that residential instability also triggers (re)emigration; highly internal mobile migrants are more likely to leave the country after naturalization. Among the factors that contribute to reducing the likelihood of (re)emigration, the retaining effect of family in the form of spouses originally from and/or children born in the host country has been shown (Jensen and Pedersen, 2008; Monti, 2020). Although the family relationship between members of households is not stated in our dataset, we find evidence of a negative correlation between household size and (re)emigration. Together with the family and according to de Hoon et al.’ (2019) findings in the case of the Netherlands, the presence of a wide network of co-nationals in the host country discourages (re)emigration. These results provide the first insight into some of the conditions and triggers for subsequent migration, which contributes to enriching and informing the design of public policies on these subjects. At the theoretical level, they illustrate a series of factors involved in the potential deployment of the recently acquired “mobility capital” (Moret, 2017) after accessing Swiss citizenship.

## Figures and Tables

**Fig. 3:**
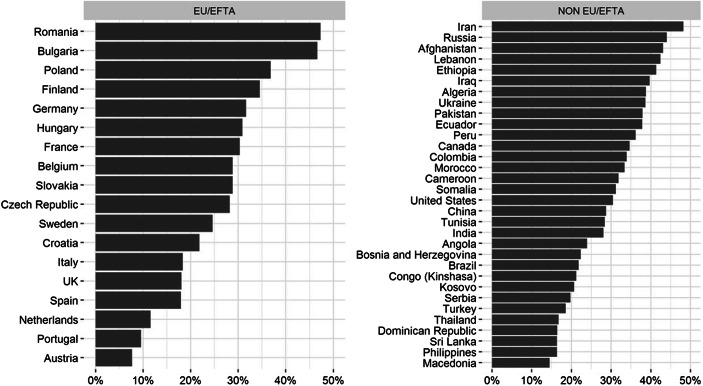
Naturalization rate by country of previous nationality, 2011–2017, top 50 countries. Source: Own elaboration based on data from the Swiss Statistical Office

## Appendix

**Table 5:** Number of emigrations following naturalization according to the procedure and the year, 2011–2017

	Ordinary		Facilitated	
	Stayed	Emigrated	Stayed	Emigrated
2011	25,845	1506	7603	615
2012	24,418	1403	6711	478
2013	24,012	1111	8112	447
2014	22,774	924	8527	384
2015	30,151	980	9028	279
2016	32,767	825	8875	184
2017	35,004	284	9326	79
Total	194,971	7033	58,182	2466

Source: Own elaboration based on data from the Swiss Statistical Office

**Table 6:** Naturalization by type in absolute and relative terms, 2011–2017

Year	Ordinary	Facilitated	Ordinary	Facilitated
2011	1745	627	2.0%	0.7%
2012	2464	498	2.8%	0.6%
2013	2835	516	3.2%	0.6%
2014	2610	520	2.9%	0.6%
2015	2877	453	3.2%	0.5%
2016	2613	388	2.9%	0.4%
2017	2162	396	2.4%	0.4%
Total	17,306	3398	19.5%	3.8%

Source: Own elaboration based on data from the Swiss Statistical Office

**Table 7:** Nationality (by groups) and country of destination of the population moving from Switzerland, 2011–2017

	Naturalized		Non-naturalized	
Group	Top destinations	N	Top destinations	N
EU/EFTA	France	32	France	107
	US	22	US	91
	UK	15	Germany	74
	Germany	13	UK	73
	United Arab Emirates	12	Austria	42
	Singapore	11	United Arab Emirates	39
	Australia	9	Spain	30
	Monaco	6	Brazil	30
	Spain	6	Singapore	30
	Thailand	5	Italy	24
NON-EU/EFTA	France	52	Kosovo	34
	UK	34	US	33
	Germany	23	France	27
	US	15	Germany	23
	Canada	9	UK	19
	United Arab Emirates	9	Canada	18
	Turkey	7	Spain	14
	Australia	6	United Arab Emirates	13
	Portugal	5	Singapore	13
	China	5	Serbia	12

Source: Own elaboration based on data from the Swiss Statistical Office

## Footnotes

1 EU/EFTA: the EU28, Iceland, Norway, the Principality of Liechtenstein and Switzerland

2 The Agreement on the Free Movement of Persons (AFMP) was concluded in 1999 between Switzerland and the European Union. It came into force in June 2002 and aims to facilitate the conditions of residence and work of EU/EFTA and Swiss citizens on the territory of the participating states (State Secretariat for Migration 2021).

3 It should be noted that the naturalization criteria mentioned were applied for the period under study (2011–2017). The legal framework has since changed: a new Federal Act on Swiss Citizenship as well as a Nationality Ordinance came into force in early 2018.

4 Because Switzerland is a party to the Refugee Convention of 1951, which states that naturalization of refugees must be facilitated, the criteria for language skills (that are part of the assessment of the level of integration into Swiss society) are expected to be relaxed for recognised refugees (State Secretariat for Migration, 2013, p. 24).
